# Location, Location, Location: How Position Affects Gene Expression in the Nucleus

**DOI:** 10.1371/journal.pbio.0030106

**Published:** 2005-03-01

Control of gene expression plays a role in determining cell fate, differentiation, and the maintenance of specific cell lineages. In the absence of regulation, aberrant gene expression can lead to developmental defects and disease. As a result, gene expression is highly regulated and that regulation takes many forms. Control mechanisms may be specific to one gene or operate on a gross chromosomal level, ultimately ensuring that genes are expressed at the right time, in the right place.

It is only in the run-up to and during cell division that chromosomes take on the condensed form that enables them to be recognized as discreet structures. During the rest of the cell cycle, interphase chromosomes exist in a relaxed state that at first glance looks like an unraveled ball of wool floating randomly about the nucleus. But a closer look reveals that they are in fact non-randomly organized and compartmentalized, and these groupings have functional ramifications for how genes are expressed or silenced (repressed).[Fig pbio-0030106-g001]

**Figure pbio-0030106-g001:**
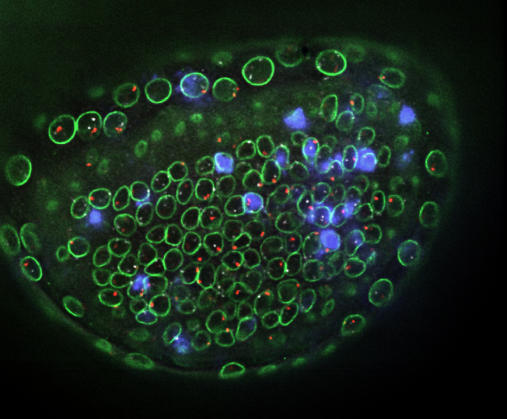
Assessing gene expression and gene location in single cells

What is actually visible is chromatin, a combination of naked DNA and proteins that associate with it. It can exist in two forms: euchromatin and heterochromatin. Actively expressed chromosomal regions (loci) are predominantly located within euchromatin, while loci within heterochromatic regions are silenced. Genetic and cytological evidence indicates that interaction between euchromatic genes and heterochromatin can cause gene silencing. Getting a gene into position for such an interaction may be achieved in two ways. The first is by changing the gene's position on the chromosome to bring it very close to expanses of centromeric heterochromatin, thereby increasing the likelihood for interaction. The second is by changing the position of a section of heterochromatin to place it close to a euchromatic gene. The small regions of heterochromatin involved in this second process seem sufficient to mediate long-range interactions between the affected gene and the larger heterochromatic regions near the centromere, but not so large or powerful as to mediate silencing by themselves. In this issue, Brian Harmon and John Sedat study the functional consequences of long-range chromosomal interactions—consequences that have been inferred in several different organisms but until now have not been analyzed on a cell-by-cell basis or directly verified.

Several Drosophila fruitfly mutants have been identified that exhibit cells in the same organ with varied phenotypes (appearance), though their genotypes (DNA instructions) are the same. This occurs through a phenomenon known as position-effect variegation, in which the expression of variegating genes is determined by their position on the chromosome relative to regions of heterochromatin. Working with fruitflies, the authors labeled three variegating genes and areas of heterochromatin with fluorescent probes and visualized expression of the affected genes in tissues where they are normally expressed. Silenced genes, they discovered, are far closer to heterochromatin than expressed genes, indicating that silenced genes interact with heterochromatin while expressed genes do not.

This study of interactions between a gene and heterochromatin in single cells illustrates unequivocally a direct association between long-range chromosomal interactions and gene silencing. The novel cell-by-cell analysis paves the way for further analysis of this phenomenon and will lead to a greater insight into the understanding and functional significance of nuclear architecture.

